# Reconfigurable terahertz metasurfaces coherently controlled by wavelength-scale-structured light

**DOI:** 10.1515/nanoph-2021-0501

**Published:** 2021-11-11

**Authors:** Kamalesh Jana, Emmanuel Okocha, Søren H. Møller, Yonghao Mi, Shawn Sederberg, Paul B. Corkum

**Affiliations:** Department of Physics, University of Ottawa, Advanced Research Complex (ARC) 25 Templeton Street Ottawa, Ottawa, ON, K1N 6N5, Canada

**Keywords:** coherent control, metasurfaces, optoelectronics, structured light, terahertz radiation, ultrafast optics

## Abstract

Structuring light–matter interaction at a deeply subwavelength scale is fundamental to optical metamaterials and metasurfaces. Conventionally, the operation of a metasurface is determined by the collective electric polarization response of its lithographically defined structures. The inseparability of electric polarization and current density provides the opportunity to construct metasurfaces from current elements instead of nanostructures. Here, we realize metasurfaces using structured light rather than structured materials. Using coherent control, we transfer structure from light to transient currents in a semiconductor, which act as a source for terahertz radiation. A spatial light modulator is used to control the spatial structure of the currents and the resulting terahertz radiation with a resolution of 5.6±0.8 μm , or approximately λ/54 at a frequency of 1 THz. The independence of the currents from any predefined structures and the maturity of spatial light modulator technology enable this metasurface to be reconfigured with unprecedented flexibility.

## 1 Introduction

The optical response of matter is primarily governed by its electric polarization. Controlling electric polarization enables light–matter interaction to be engineered, and for the effective optical response to mimic materials with natural or artificial properties. A metasurface consists of a planar ensemble of deeply subwavelength structures or films that are designed to sculpt the spatial structure of the field vectors, intensity, or phase fronts of the incident light [1, 2]. Among numerous functionalities, metasurfaces are used to create flat lenses [3], [4], [5], [6], [7], superlenses [8, 9], waveplates [10], [11], [12], [13], [14], phase plates for generating vortex beams [15], [16], [17], and elements for phase front control [18, 19].

Despite the advanced optical capabilities offered by metasurfaces, the inherent complexity of simulating and fabricating large ensembles of subwavelength structures limits their proliferation. Moreover, once fabricated, the function and operational parameter space of the metasurface is fixed, limiting its utility. In many applications it is desirable to actively control the properties of a metasurface. Homogeneous adjustment of metasurfaces is possible by tuning or modulating the optical properties of the fabricated structures or their supporting substrate through approaches including the thermo-optic effect, photodoping, and phase-change materials [20, 21]. Inhomogeneous tuning of metasurfaces, or reconfigurability, is much more challenging and has primarily been demonstrated at microwave frequencies. Reconfigurability of microwave metasurfaces is achieved by connecting active electronic components, such as varactors, diodes or transistors, to each meta-atom comprising the metasurface. A voltage applied to the electronic component in each meta-atom tunes its electromagnetic response and controls the local phase imparted to an incident microwave field [22], [23], [24], [25]. At higher frequencies, it becomes increasingly challenging to integrate electronic devices with structures comprising the metasurface.

At terahertz (THz) frequencies, structure can be transferred from an optical beam to the optical properties of a material through photodoping or a local phase change. For example, a digital micromirror device has been used to spatially structure the intensity of a λ=800 nm optical beam, which was used to exert spatial control over an insulator-to-metal phase transition in VO_2_ [26]. When a broadband THz pulse is incident on the VO_2_, the metallic regions of the material preferentially reflect the THz fields, resulting in intensity structuring of the transmitted THz fields. One promising route to reconfigurable metasurfaces at near-infrared wavelengths is based on electrically controlled phase-change materials [27]. Although voltages have been used to reconfigure specific metasurface functionalities, flexible control of individual meta-atoms has not yet been demonstrated.

Beyond their linear optical response, metasurfaces can also be used to control nonlinear optical interactions, and to shape the properties of new colors of light arising from frequency upconversion or downconversion in the metasurface. For instance, metasurfaces have been used to enhance the generation of perturbative [28, 29] or high-order harmonics [30, 31], and to achieve self-focusing of the harmonics. Similarly, optical rectification in a metasurface can control the spatiotemporal structure of THz radiation [32, 33]. Terahertz waves have found broad-ranging utility in spectroscopy, imaging, communications, and resolving femtosecond electron dynamics in semiconductors. Although the generation and detection of THz radiation have undergone vast development over the last 3 decades, devices for manipulating its spatial structure lag behind their optical frequency counterparts. One particular challenge lies in sculpting the spatial structure of the entire bandwidth of THz pulses, which can be of sub-cycle duration. Overcoming this challenge introduces the possibility to investigate space-time-coupled solutions to Maxwell’s equations such as a flying electromagnetic torus [34].

The inherent link between electric polarization and current density stimulates an alternative approach to metasurfaces: one composed of subwavelength elements of dynamic current density rather than subwavelength scattering elements. Coherent control of dynamic semiconductor currents is possible when bichromatic laser fields at the appropriate wavelengths excite the semiconductor [35], [36], [37], [38], [39], [40], [41], [42], [43]. In its conventional implementation, a fundamental laser pulse (ω) excites a conduction band population via two-photon absorption (TPA), and its second harmonic (2ω) is linearly absorbed by the semiconductor, exciting a conduction-band population at the same point in *k*-space. Quantum interference enables the relative phase between the two laser fields, $\Delta \varphi_{\omega ,2\omega }$ , to control momentum asymmetry in the conduction band population. In this manner, adjustment of $\Delta \varphi_{\omega ,2\omega }$ controls the amplitude and sign of currents injected into the semiconductor. These currents turn on within the timescale of the laser pulse durations (i.e., femtoseconds) and relax through the emission of longitudinal optical phonons and collisional processes lasting roughly 200–300 fs. The resulting current radiates a THz electromagnetic impulse. Conventionally, coherent control is performed using the lowest-order Hermite–Gaussian laser mode. As a result, the currents introduced to the semiconductor by different spatial regions of the laser mode all flow in the same direction.

Applying structured light beams to coherent control in a semiconductor allows the structure from optical frequency light to be transferred to currents and THz radiation. Previously, cylindrical vector beams have been applied to coherent control, and the excitation of ring currents and their accompanying magnetic fields was investigated numerically and experimentally [44, 45]. Because coherent control is sensitive to the relative phase between two laser fields, it is also interesting to introduce phase structure to one or both of the laser beams to spatially address currents in the semiconductor. Sculpting the phase fronts of one of the beams using a spatial light modulator (SLM) provided a means to independently program the vector of hundreds of current elements excited in the semiconductor [46].

The lightwave diffraction limit determines the smallest current elements that can be controlled in a bulk, unstructured semiconductor. Control of current elements with dimensions on the order of the wavelength of the exciting light introduces deeply subwavelength structure to the radiated THz fields. Here, we demonstrate the use of subwavelength current elements instead of scattering structures as a metasurface for THz radiation. Using an SLM, we control hundreds of current elements with a spatial resolution of 5.6±0.8 μm , and provide a measurement of the THz electric field waveform radiated from GaAs. While conventional metasurfaces are fixed in physical form, structuring light with an SLM enables this THz metasurface to be reconfigured with extreme flexibility. To demonstrate the versatility of this platform, we control current arrangements that resemble metasurface structures, including a magnetic-field lattice, a bowtie antenna, and a bull’s eye grating.

## 2 Experimental set-up

**Figure 1:**
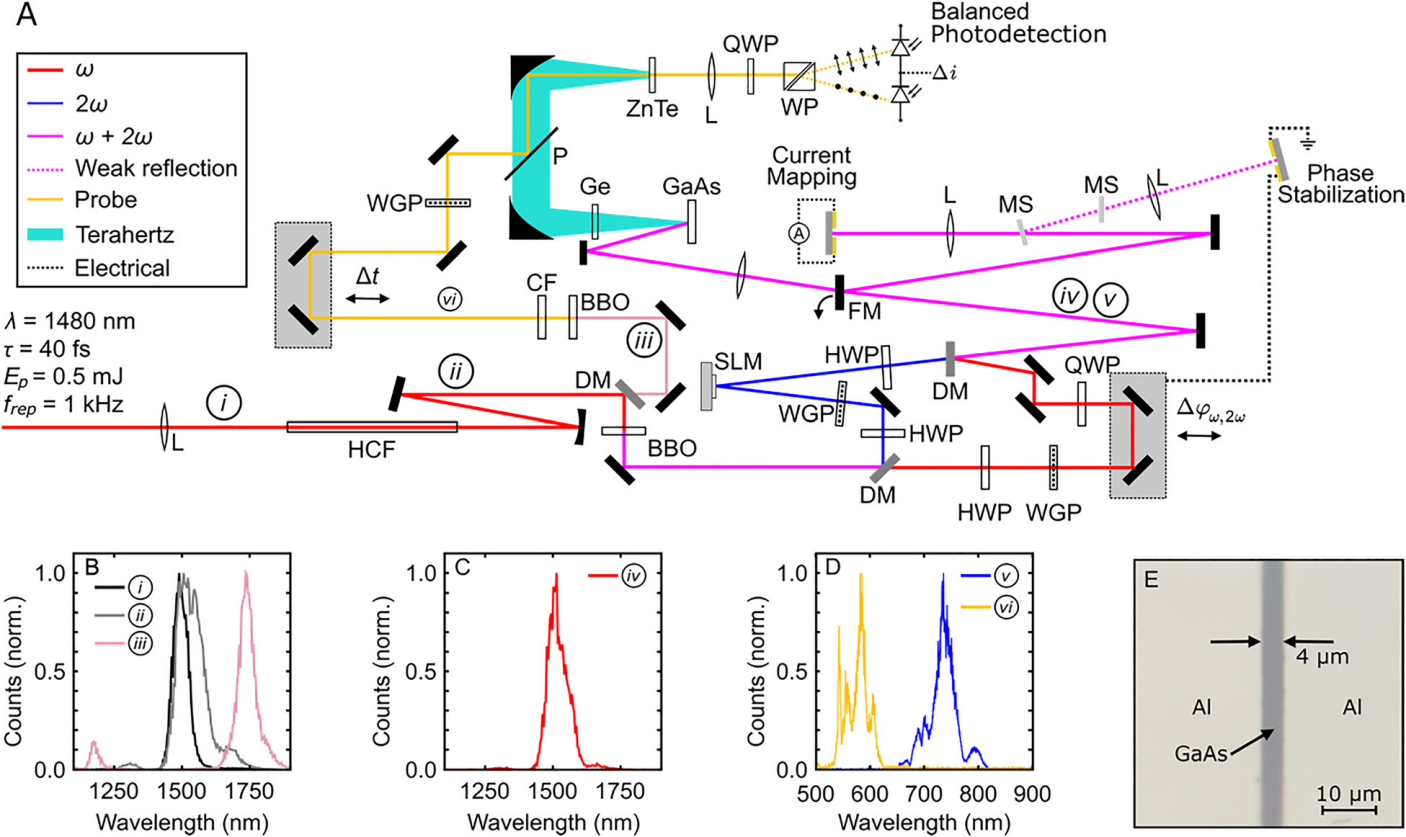
Experimental set-up. (A) The signal beam from an optical parametric amplifier (OPA) is spatially filtered and spectrally broadened using a hollow core fiber (HCF) in ambient air. A dichroic mirror (DM) transmits the spectral wings and reflects the primary spectral content. The reflected light is frequency-doubled using a β- barium borate (BBO) crystal. A two-color interferometer is used to control the relative phase between these two colors of light and impart structure to the 2ω beam using a SLM. The blue spectral tail of the broadened spectrum is frequency-doubled to generate a probe pulse for electro-optic sampling (EOS). A flipper-mirror (FM) is used to direct the bichromatic fields to either an optoelectronic detector for spatio-vectorial current mapping or a GaAs substrate for terahertz generation and EOS. L: Lens; HWP: half-wave plate; WGP: wire grid polarizer; QWP: quarter-wave plate; MS: microscope slide; P: pellicle; WP: Wollaston prism; CF: color filter. (B) Measurement of the OPA spectrum before (black) and after (gray) the HCF. The spectral wings transmitted through the DM are plotted in light pink. (C) The fundamental (ω) laser pulse after the two-color interferometer. (D) The 2ω laser pulse after the two-color interferometer (blue) and the probe pulse for EOS (yellow). (E) Optical micrograph of the active component of the optoelectronic detector used for spatio-vectorial current mapping. The substrate consists of a 1.2- μm -thick LT-GaAs film grown on a GaAs substrate. Two large aluminum electrodes separated by a 4 -μm gap are deposited onto the LT-GaAs surface.

The experimental set-up is shown in Figure 1A. An OPA operating at a 1 kHz repetition rate is tuned such that its signal beam is at λ=1480 nm and has a pulse energy of 0.5 mJ. This beam is spatially filtered by coupling it into a HCF with a core diameter of 350 μm , situated in ambient air. Due to the optical nonlinearity of air, the spectrum of the pulse incident on the fiber becomes broadened and red-shifted, as shown in Figure 1B. The spatially filtered beam is transmitted through a BBO nonlinear crystal phase-matched for second-harmonic generation.

The two colors of light are diverted into separate optical paths using a DM. The energy of each beam is controlled independently using a half-wave plate and a wire grid polarizer. The 2ω beam is reflected from an SLM (Hamamatsu X10468-02) at near-normal incidence, which imparts a pixelated phase front to the beam. The ω beam is reflected from two mirrors fixed to a delay stage with a piezoelectric actuator which allows $\Delta \varphi_{\omega ,2\omega }$ to be precisely controlled. A quarter-wave plate (QWP) is used to adjust the polarization of the ω beam from linear to circular. The two beams are combined collinearly using a second DM. The spectrum of the ω and 2 ω beams after the interferometer are shown in Figure 1C and D, respectively.

This two-color interferometer is used to coherently control currents in low temperature grown gallium arsenide (LT-GaAs). The band-gap energy $\left(E_{\text{g}}=1.55\text{ eV}\right)$ of LT-GaAs enables a conduction-band population to be excited via direct absorption using the 2ω beam and TPA using the ω beam. A mirror on a flipper mount is used to select one of two measurements.

In the first measurement we use EOS to record the electric-field waveform of THz radiation emitted from LT-GaAs. When the flipper mount position is set such that the laser pulses do not reflect from the mirror, the pulses are focused onto the backside of an LT-GaAs substrate using a biconvex lens with f=200 mm , where they generate currents. A THz impulse is radiated in both the forward and backward directions. Critically, a fraction of the ω beam propagates through the GaAs substrate and generates charge carriers via TPA. This free-carrier plasma suppresses THz propagation through the GaAs substrate. The THz impulse radiated into air is collected using an off-axis parabolic mirror. A 0.5-mm-thick uncoated germanium substrate absorbs the residual ω and 2 ω pulses, but transmits a fraction of the THz pulse.

To generate a probe pulse that is spectrally isolated from the ω and 2 ω pulses, we introduce a DM to the OPA beam shortly after it has been broadened in the HCF. This mirror transmits the blue and red tails of the broadened spectrum. We use a second BBO crystal to frequency double the blue tail at λ=1180 nm , obtaining a probe pulse at λ=590 nm , as shown in Figure 1D. A narrow bandpass color filter blocks the residual near-infrared light. After being transmitted through a wire grid polarizer, the probe pulse is combined collinearly with the THz pulse using a pellicle and, using a second off-axis parabolic mirror, they are both focused into a 1-mm-thick ZnTe crystal for EOS. Two mirrors mounted to a motorized delay stage control the arrival time of the probe pulse within the THz waveform. After loosely refocusing the probe pulse, its change in polarization in response to the instantaneous THz field is detected using a conventional scheme consisting of a QWP, Wollaston prism, and balanced photodetector. A lock-in amplifier (Stanford Research Systems SR830) reads out the EOS signal.

In the second measurement, the flipper mount is positioned such that the ω and 2 ω pulses reflect from the mirror. A biconvex lens with f=200 mm images the phase pattern of the SLM onto an LT-GaAs optoelectronic detector. This imaging configuration simultaneously demagnifies the pixelated phase structure imparted to the beam in the SLM plane by a factor of 5. The SLM has a pixel pitch of 20 μm , and therefore in this configuration it is possible to control current elements with dimensions as small as 4 μm , or approximately 2.7 wavelengths of the fundamental light.

In order to detect currents excited in the LT-GaAs, we deposit two large aluminum electrodes separated by a 4-μm gap onto the semiconductor. An optical micrograph of the fabricated device is shown in Figure 1E. This 4-μm gap limits the spatial extent of light that can enter the semiconductor and drive currents in one dimension. An optical mask with two large electrodes in the orthogonal orientation is fixed on top of the LT-GaAs substrate, and the metallic structures of the combined substrates form an aperture, which defines the spatial extent of light that can enter the detector. Due to differences in the fabrication process, the metallic features of the mask are separated by a smaller gap of approximately 2.5 μm . Although the aperture is rectangular, it is still effective for measuring and mapping currents with a resolution on the order of 4 μm .

A weak reflection from a borosilicate microscope slide is focused onto a second LT-GaAs optoelectronic detector, which is used as a meter for $\Delta \varphi_{\omega ,2\omega }$ . A software-based feedback loop can be used to stabilize the current in this detector by adjusting the position of the piezoelectric delay stage. This feedback scheme compensates for slow drifts in $\Delta \varphi_{\omega ,2\omega }$ that influence the measurements.

## 3 Results

**Figure 2:**
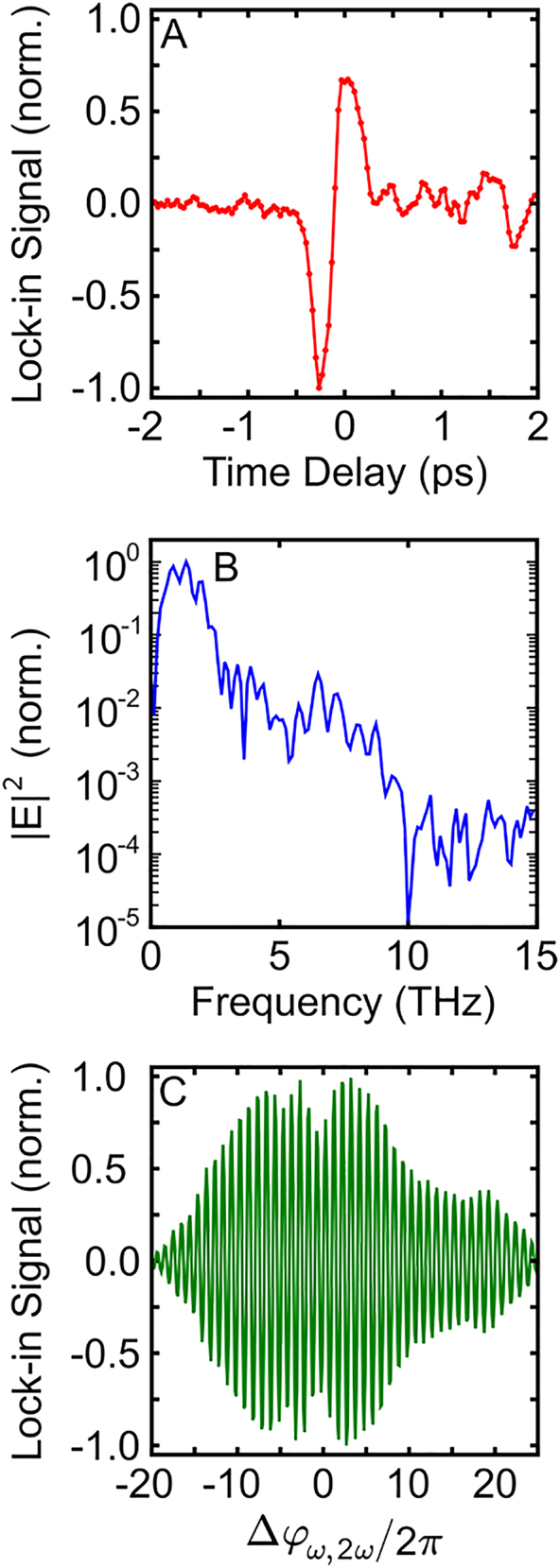
EOS of terahertz pulses. (A) Recorded electro-optic signal versus time-delay of the probe pulse. (B) Power spectral density of the waveform plotted in (A). (C) EOS signal at a fixed probe delay as $\Delta \varphi_{\omega ,2\omega }$ is adjusted.

First, we measure the THz waveform radiated from dynamic currents in GaAs. In this measurement, both the ω and 2 ω pulses have linear polarization, and no phase pattern is introduced to the SLM. This results in a linearly polarized THz pulse that does not contain spatial structure. An exemplary EOS scan of the THz waveform is shown in Figure 2A, and the power spectral density of this waveform is plotted in Figure 2B. Notably, ZnTe has a weak electro-optic response at frequencies greater than 5 THz, and we anticipate that the measurement does not capture the full bandwidth of the THz pulse. Keeping the probe pulse time delay fixed at the peak of the THz waveform, we adjust $\Delta \varphi_{\omega ,2\omega }$ and measure its influence on the THz electric field, plotted in Figure 2C. The oscillations between positive and negative signal confirm that we coherently control the amplitude and polarity of the THz waveform.

We subsequently introduce a phase pattern to the SLM for the purpose of structuring currents and THz radiation from LT-GaAs. Although, a full spatio-temporal characterization of the THz fields would provide a rigorous demonstration of the metasurface, here we focus on characterizing the source of the THz radiation, i.e., the dynamic semiconductor currents. We note that a dynamic current density acts as a source for electromagnetic radiation via the Ampère–Maxwell law:

$$\nabla \times H=J+\frac{\partial D}{\partial t},$$

where ***H*** is the magnetic field, ***J*** is the current density, ***D*** is the electric displacement field, and *t* is the time. Therefore, characterizing current density on a deeply subwavelength scale provides direct insight into the radiated THz fields.

**Figure 3:**
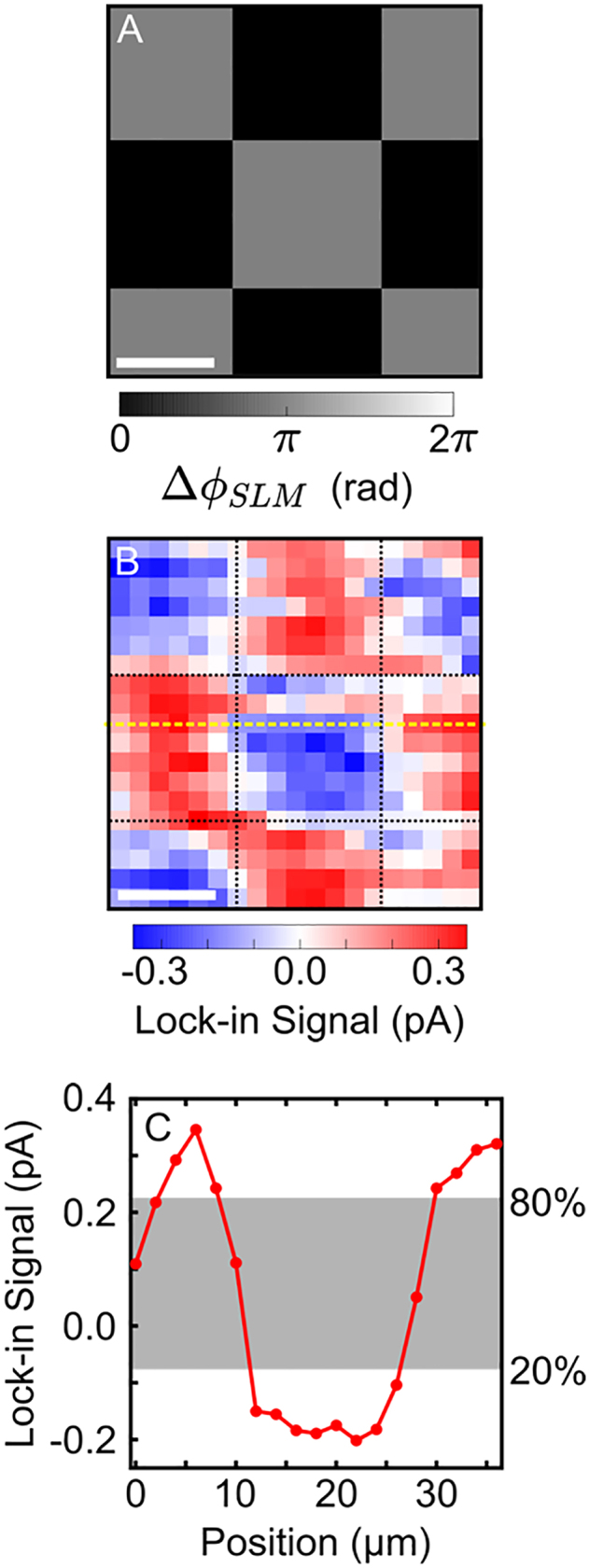
Wavelength-scale control and measurement of currents. (A) Checkerboard SLM pattern used to control micron-scale currents. The scale bar represents 50 μm . (B) Spatial mapping of the *x*-component of the current measured using the optoelectronic detector. The scale bar represents 10 μm . (C) Plot of the yellow line-out shown in (B). The gray shaded region portrays the region over which the current signal grows from 20 to 80% of its peak-to-peak amplitude.

To determine the resolution with which we can control and measure currents, we introduce a checkerboard phase pattern to the SLM (Figure 3A). The polarization of the ω beam is converted to circular to enable full control of the current direction in the transverse plane by adjustment of $\Delta \varphi_{\omega ,2\omega }$ . Keeping $\Delta \varphi_{\omega ,2\omega }$ fixed, we scan the detector across the optical beams in a 2-dimensional raster grid with 2 μm steps and measure the current at each grid position. A complete description of the experimental techniques used to control and measure the spatio-vectorial current arrangements can be found in refs. [44, 45].

A spatial mapping of the *x*-component of the detected current is shown in Figure 3B, where it is evident that the checkerboard pattern introduced to the SLM is transferred to the semiconductor currents. An exemplary line-out of this scan is plotted in Figure 3C, where the shaded box represents the region in which the edge between positive and negative checker squares grows from 20–80%. We use the 20–80% edge width as a measure of the combined resolution of our current control and detection schemes. By performing a statistical analysis on the entire scan shown in Figure 3B, we estimate the combined resolution to be 5.6±0.8 μm . Several factors that could degrade the ideal 4 μm resolution include diffraction from the optical mask, imperfect imaging of the SLM pattern, imperfect wavefront matching between the ω and 2 ω beams, and imperfect overlap of the detector pixel with the imaged SLM pixels resulting in averaging of currents excited by adjacent SLM pixels. Nevertheless, we conclude that both the phase imaging configuration and the LT-GaAs current detector are close to optimized.

Next, we manipulate and measure the vectorial arrangement of the currents and demonstrate patterns that resemble metasurfaces. The split-ring resonator is a common building block for left-handed metamaterials that enables an incident electric field to excite a magnetic resonance in the structure, i.e., an oscillating ring current is excited. While a gap is required in a split-ring resonator to excite a magnetic mode, no such gap is required if the currents are generated directly. To this end, we excite a 2×2 lattice of ring currents that can be extended to larger arrays resembling a metasurface.

**Figure 4:**
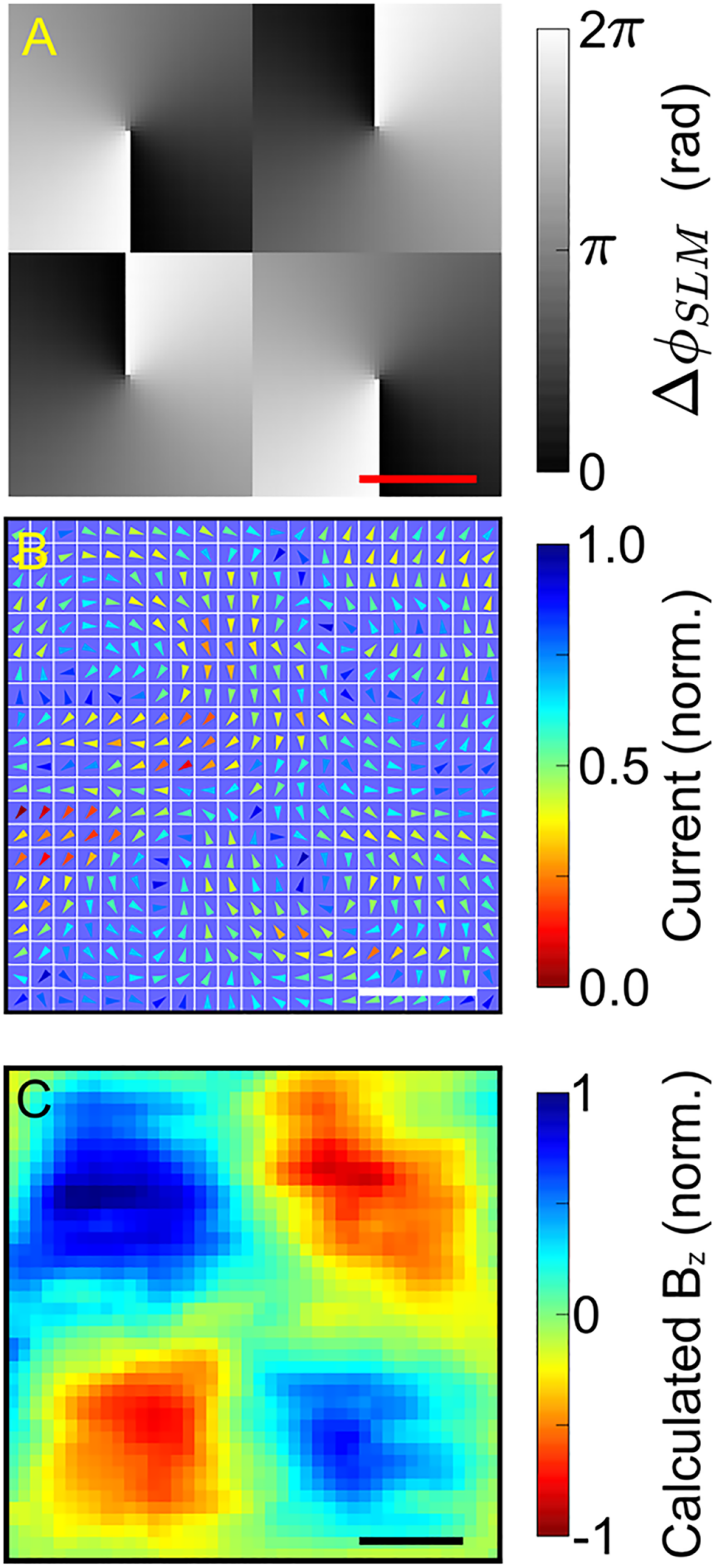
Control of magnetic field lattices. (A) The SLM pattern used to control magnetic fields. Each conical gradient controls a ring current and magnetic field at the corresponding lattice site. The scale bar represents 100 μm . (B) Measured spatio-vectorial current arrangement, where a 2×2 array of ring currents is resolved. The scale bar represents 20 μm . (C) A calculated snapshot of the magnetic field resulting from the dynamic current arrangement plotted in (B). The snapshot is calculated 100 fs after the laser pulse excitation. The scale bar represents 20 μm .

The SLM pattern used to excite a lattice of ring currents with alternating direction is shown in Figure 4A, where a conical gradient phase structure at each lattice site controls a ring current. The application of an ω beam carrying intrinsic angular momentum and a 2ω beam with localized orbital angular momentum to coherent control results in the excitation of a ring current at each lattice site. The amplitude and direction of each ring current and its accompanying magnetic field can be programmed using the SLM. The spatio-vectorial current map recorded using the phase pattern in Figure 4A is shown in Figure 4B, where a step size of 4 μm is used for the measurement.

Assuming that the current is injected within the timescale of the laser pulse duration and relaxes with a characteristic timescale of $\tau_{r}=200\text{ fs}$ , we evaluate the magnetic vector potential:

$$A\left(r,t\right)=\frac{\mu_{0}}{4\pi }\int \frac{J\left(r^{\prime },t_{r}\right)}{\left|r-r^{\prime }\right|}{\text{d}}^{3}r^{\prime },$$

where r is the point in space under evaluation, $r^{\prime }$ is the point in a space at which the current density element under consideration is located, $\mu_{0}$ is the permeability of free space, $J\left(r^{\prime },t_{r}\right)$ is the dynamic current density at each point in space, $t_{r}=\frac{\left|r-r^{\prime }\right|}{c}$ is the inherent time delay before a current element at $r^{\prime }$ at a particular instant influences the vector potential at r , and c is the speed of light. The magnetic field arrangement can then be calculated as B(r,t)=∇×A(r,t) . Using this approach, the magnetic field lattice excited by the measured ring current elements 100 fs after the laser excitation is shown in Figure 4C. In this scan, the separation between adjacent lattice sites is approximately 35 μm and the magnetic field at each lattice site has a full width at half-maximum of approximately 20 μm .

**Figure 5:**
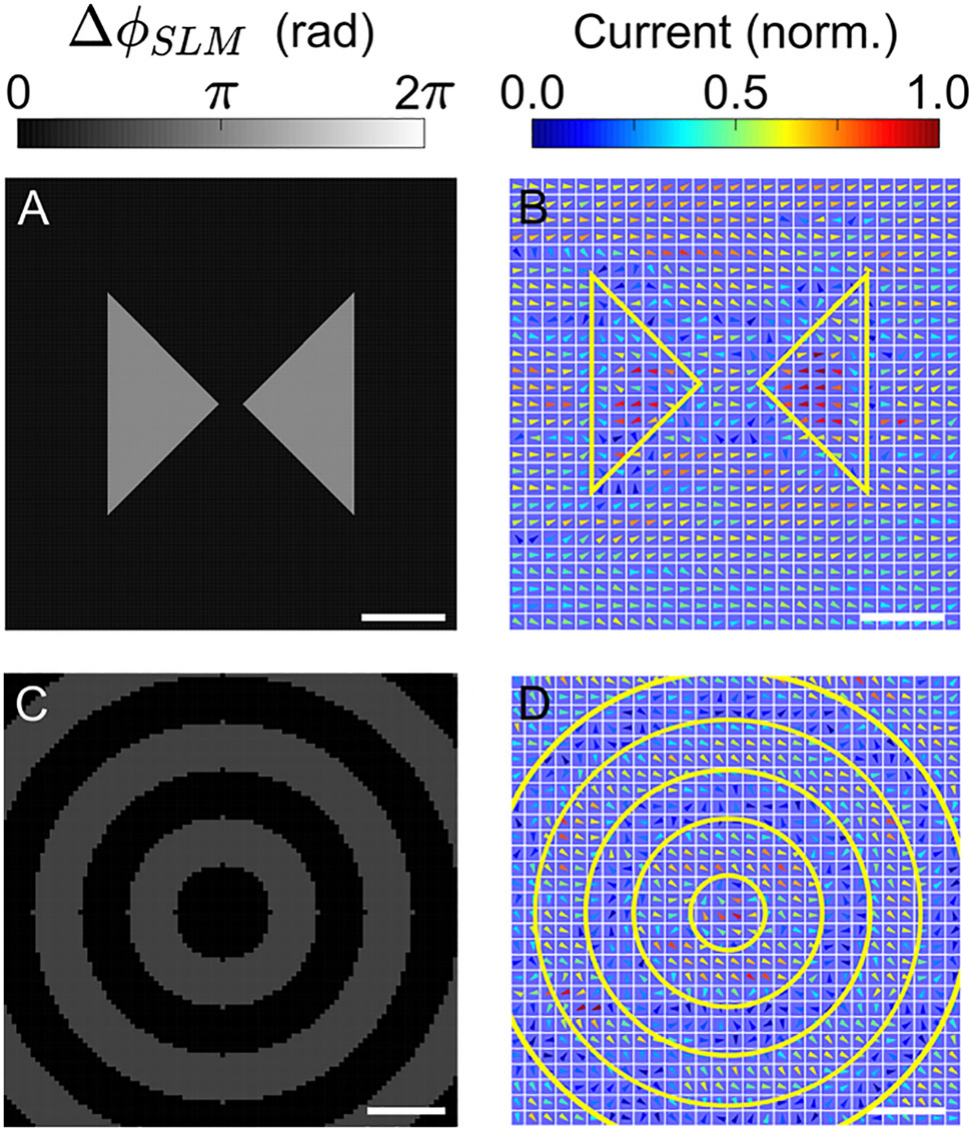
Structured currents for metasurfaces. (A) The SLM pattern used to excite a dynamic current resembling a bowtie antenna in the LT-GaAs. The scale bar represents 100 μm . (B) The current arrangement resulting from the SLM pattern in (A). The scale bar represents 20 μm . (C) An SLM pattern resembling a bull’s eye structure. The scale bar represents 100 μm . (D) The current arrangement resulting from the central rings of the SLM pattern in (C). The scale bar represents 20 μm . The yellow lines indicate the approximate location of phase jumps in the respective SLM patterns.

Antennas are used to transmit and receive electromagnetic signals, to generate THz radiation using photoconductive switches, to enhance and confine light–matter interaction, and as elements of metasurfaces. One common antenna design is the bowtie antenna. Using the SLM pattern in Figure 5A, we excite a dynamic bowtie antenna for THz radiation. The measured current distribution is shown in Figure 5B. The total length of this antenna is approximately 65 μm corresponding to λ/2 of 1 THz if the effective refractive index of the antenna’s environment is $n_{\text{eff}}=2.2.$ The shape of the antenna can be adjusted with great flexibility using the SLM phase to control its radiation pattern. In contrast to conventional antennas, where the direction of currents within the antenna are determined by electric fields, currents within this dynamic antenna can be injected in any transverse direction. These antennas can be viewed as a new class of “smart antenna.” A lattice of such antennas could be used as an antenna array or as a metasurface.

Although the injected currents relax within ∼100 fs, the electron-hole plasma remains for ∼1 ns. This presents the opportunity to use currents to initiate plasmons, which are subsequently sustained by the semiconductor plasma. Doing so would enable spatial structure to be imparted to surface plasmon polaritons (SPP) by the incident light. To introduce this concept, we excite a collection of concentric rings of current, or a bull’s eye structure. Bull’s eye structures have been used to achieve high transmission of light incident on the bull’s eye through a sub-diffraction aperture situated at its center, an attractive feature for near-field microscopy [47, 48]. An exemplary bull’s eye SLM pattern is shown in Figure 5C and the corresponding current measurement is plotted in Figure 5D. For this proof-of-principle, the width of each ring is kept small (approximately 12 μm ); however, we note that a more realistic implementation of this concept would require larger rings with thicknesses on the order of one wavelength of the SPP. Beyond the excitation of SPPs, concentric current rings in the form of a Fresnel lens could enable self-focusing of radiated THz pulses.

## 4 Conclusions

Rather than sculpting light–matter interaction by introducing deeply subwavelength structures to a material, we use structured light to coherently control the spatio-vectorial arrangement of dynamic currents in LT-GaAs. By introducing one structured light beam to two-color coherent control, we have programmed and measured the current vector excited in LT-GaAs by hundreds of pixels of the two-color fields. The combined resolution of our current excitation and detection schemes is measured to be 5.6±0.8 μm . Each dynamic current element acts as a sub-wavelength source for THz radiation. Using EOS, we have recorded the temporal waveform of THz radiation emitted from a single dynamic current element. The programmability of the SLM enables us to reconfigure the excited currents to resemble a lattice of ring currents, a bowtie antenna, and a bull’s eye structure. The SLM used in these experiments enables the metasurface to be reconfigured at a rate of 60 Hz; however, we note the availability of SLMs with kHz refresh rates.

To facilitate a reasonable data acquisition time, we have performed measurements consisting of several hundred current elements but note that most SLMs enable phase control in millions of pixels of a laser beam. Independent control of millions of current elements – each acting as a source for THz radiation – would provide similar intricacy as conventional metasurfaces. Programmable control of THz radiation introduces the possibility to apply optimization and inverse design procedures to reconfigurable metasurfaces and enables new schemes for point-to-point communications and imaging. Beyond this, it will facilitate exploration of new space-time-coupled modes of light, non-diffracting THz beams, the use of THz fields for controlling and accelerating charged particles, and THz magnetic field impulses. Finally, we note that injecting currents via strong-field coherent control could be used to increase both the spatial resolution of the currents and the frequency of the radiated fields [49, 50].
